# Supplementary material for: Cell-Main Spectra Profile Screening Technique in Simulation of Circulating Tumour Cells Using MALDI-TOF Mass Spectrometry
Source: Cancers (Basel). 2021 Jul 27;13(15):3775. doi: 10.3390/cancers13153775 (PMC8345129; doi:10.3390/cancers13153775)
Supplement: Supplementary file 1 [file cancers-13-03775-s001.zip › cancers-1285501-supplementary.pdf]

Supplementary Materials

Cell-Main Spectra Profile Screening Technique in Simulation of Circulating Tumour Cells Using MALDI-TOF Mass Spectrometry

Wararat Chiangjong, Sebastian Chakrit Bhakdi, Noppawan Woramongkolchai, Thitinee Vanichapol, Nutkridta Pongsakul, Suradej Hongeng and Somchai Chutipongtanate

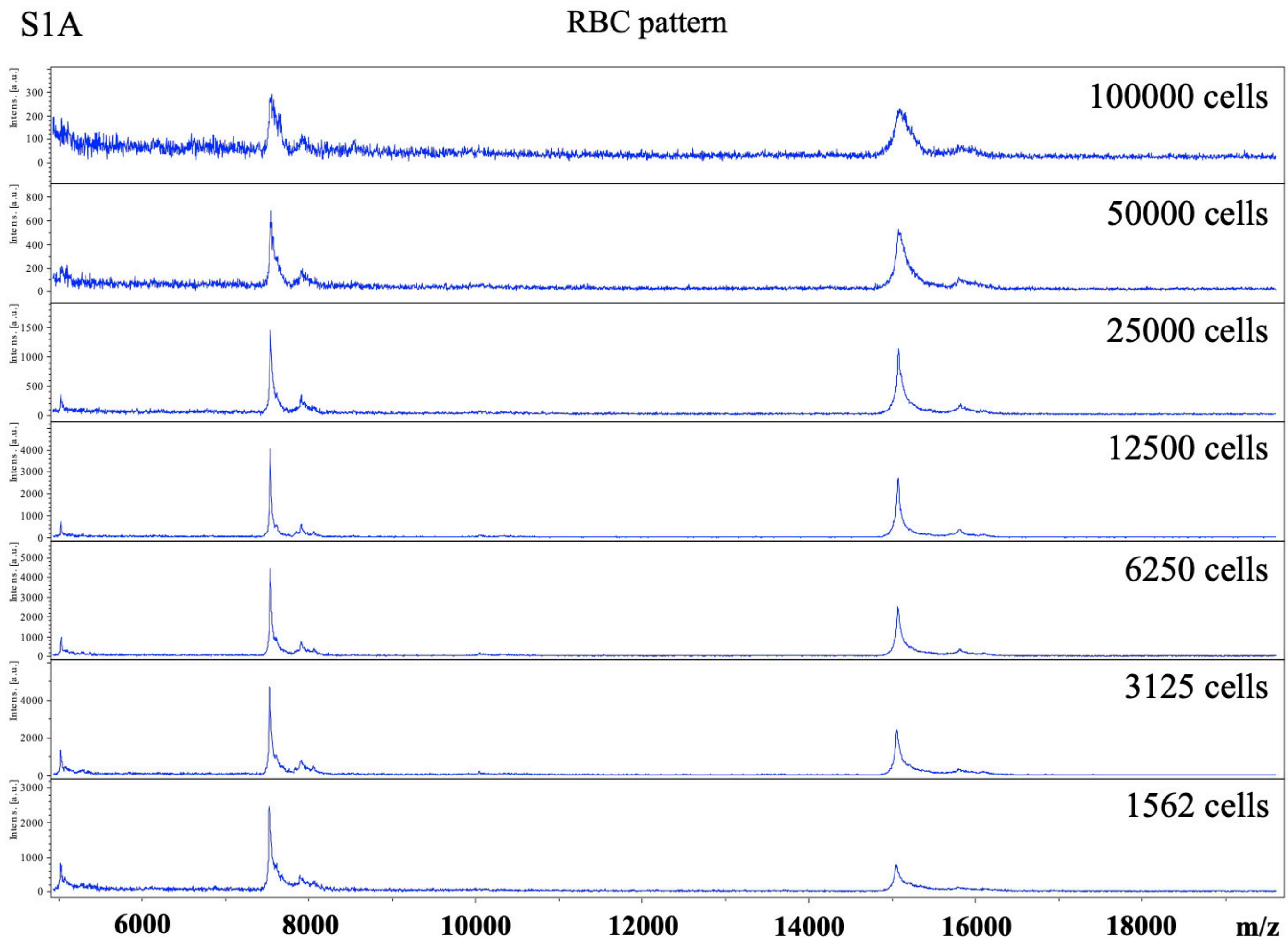

S1B

WBC pattern

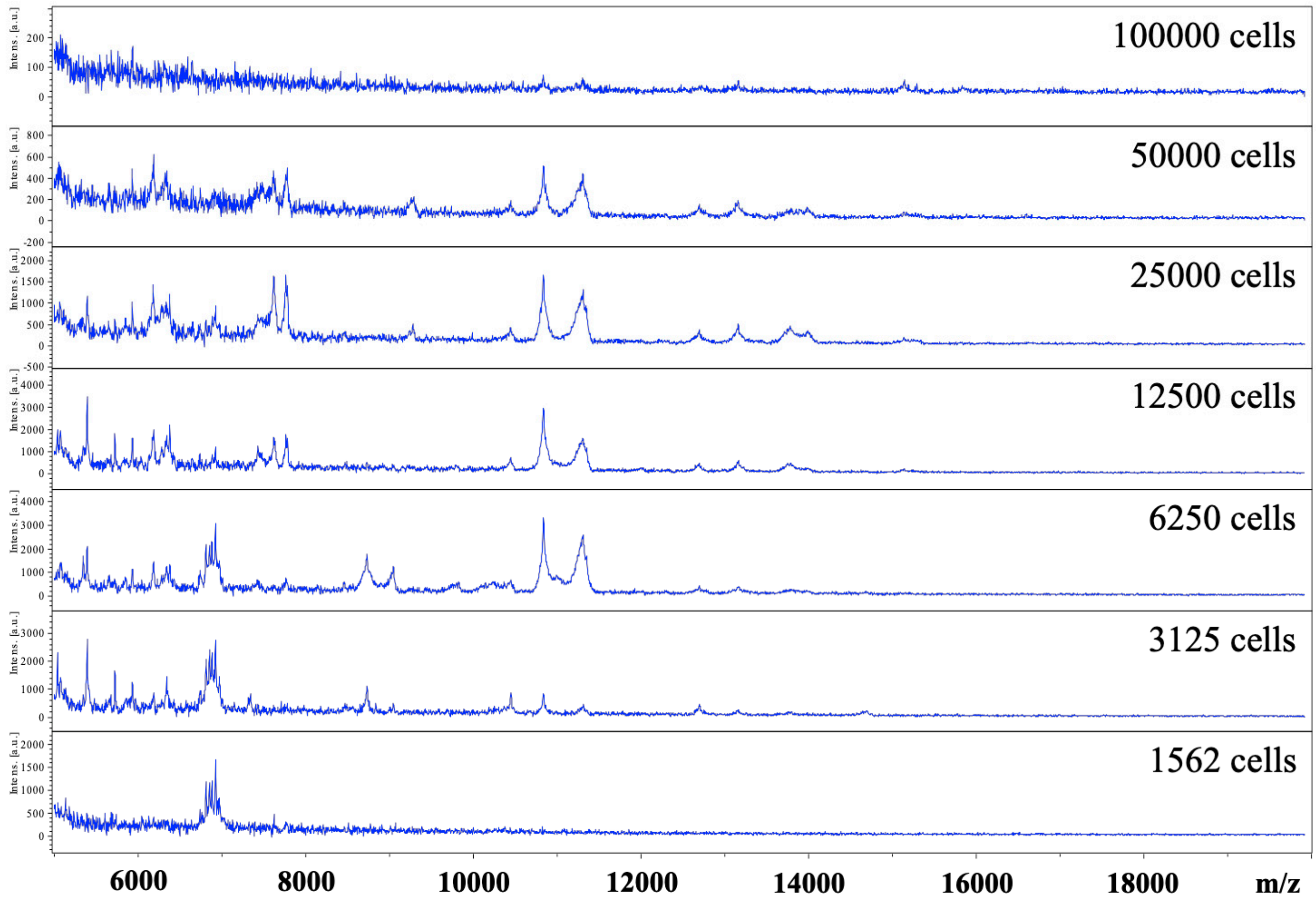

S1C

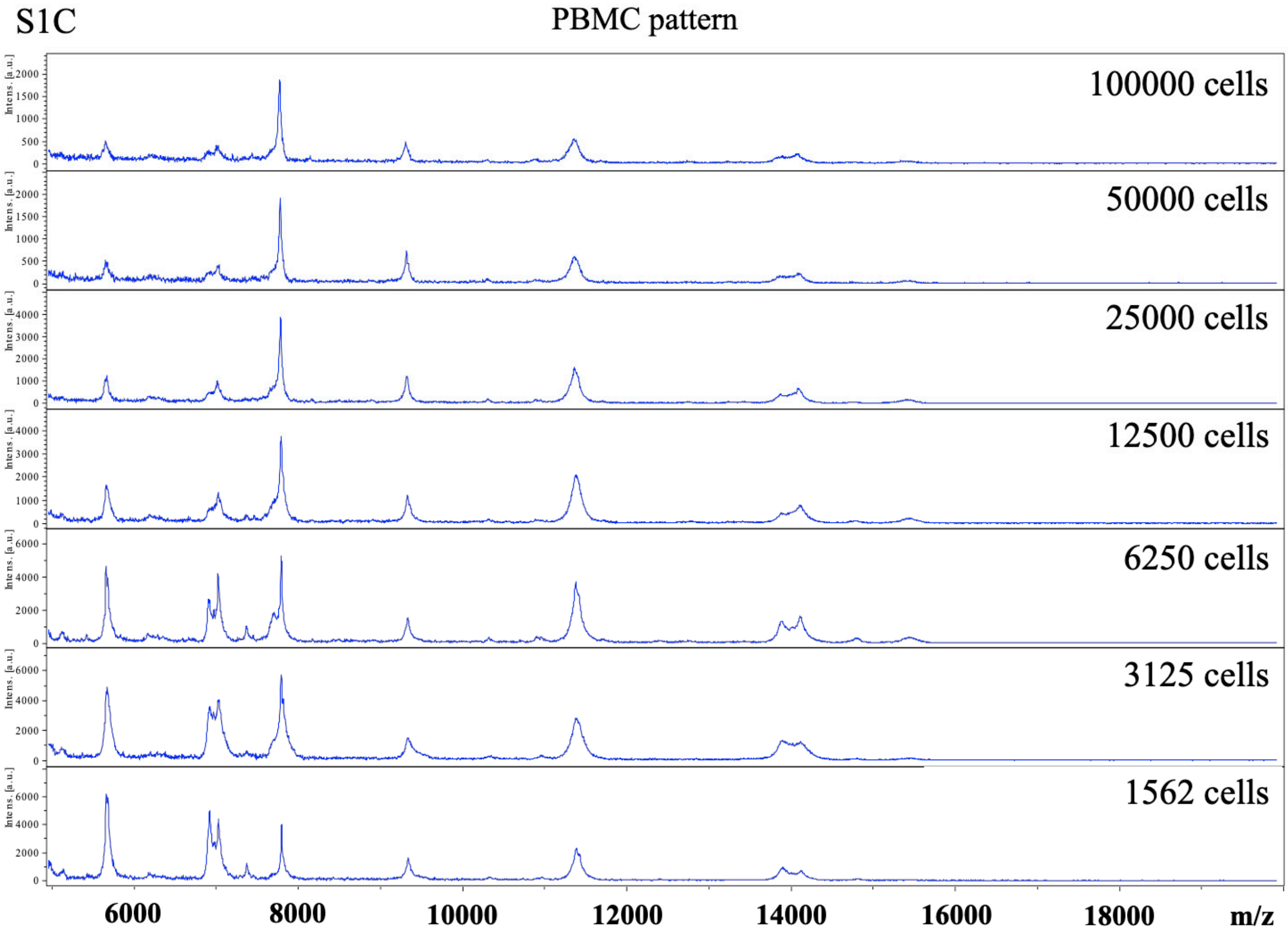
PBMC pattern
100000 cells
50000 cells
25000 cells
12500 cells
6250 cells
3125 cells
1562 cells
Intens. [a.u.]
6000
8000
10000
12000
14000
16000
18000
m/z

S1D

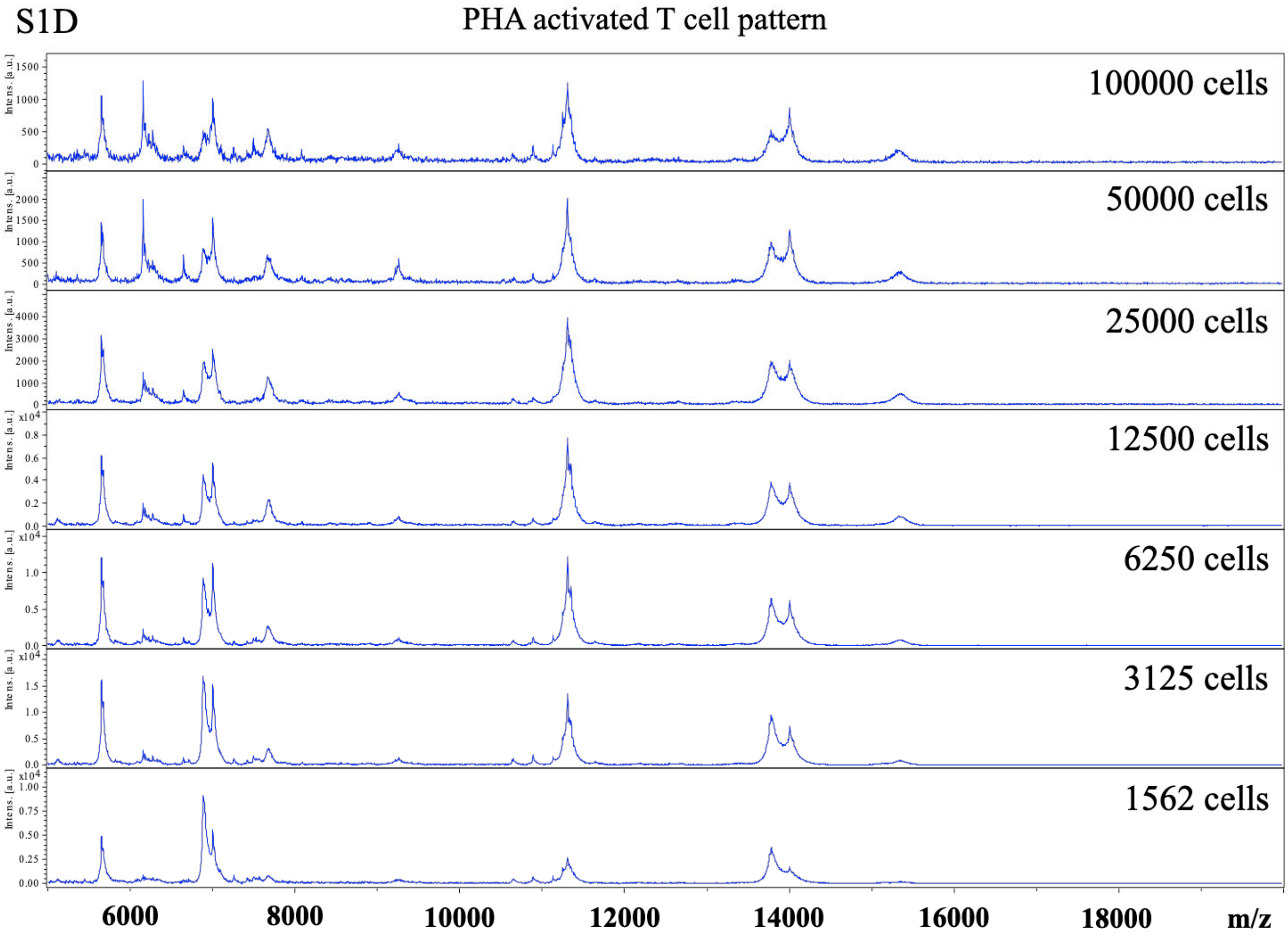
PHA activated T cell pattern
100000 cells
50000 cells
25000 cells
12500 cells
6250 cells
3125 cells
1562 cells
Intens. [a.u.]
6000
8000
10000
12000
14000
16000
18000
m/z

S1E

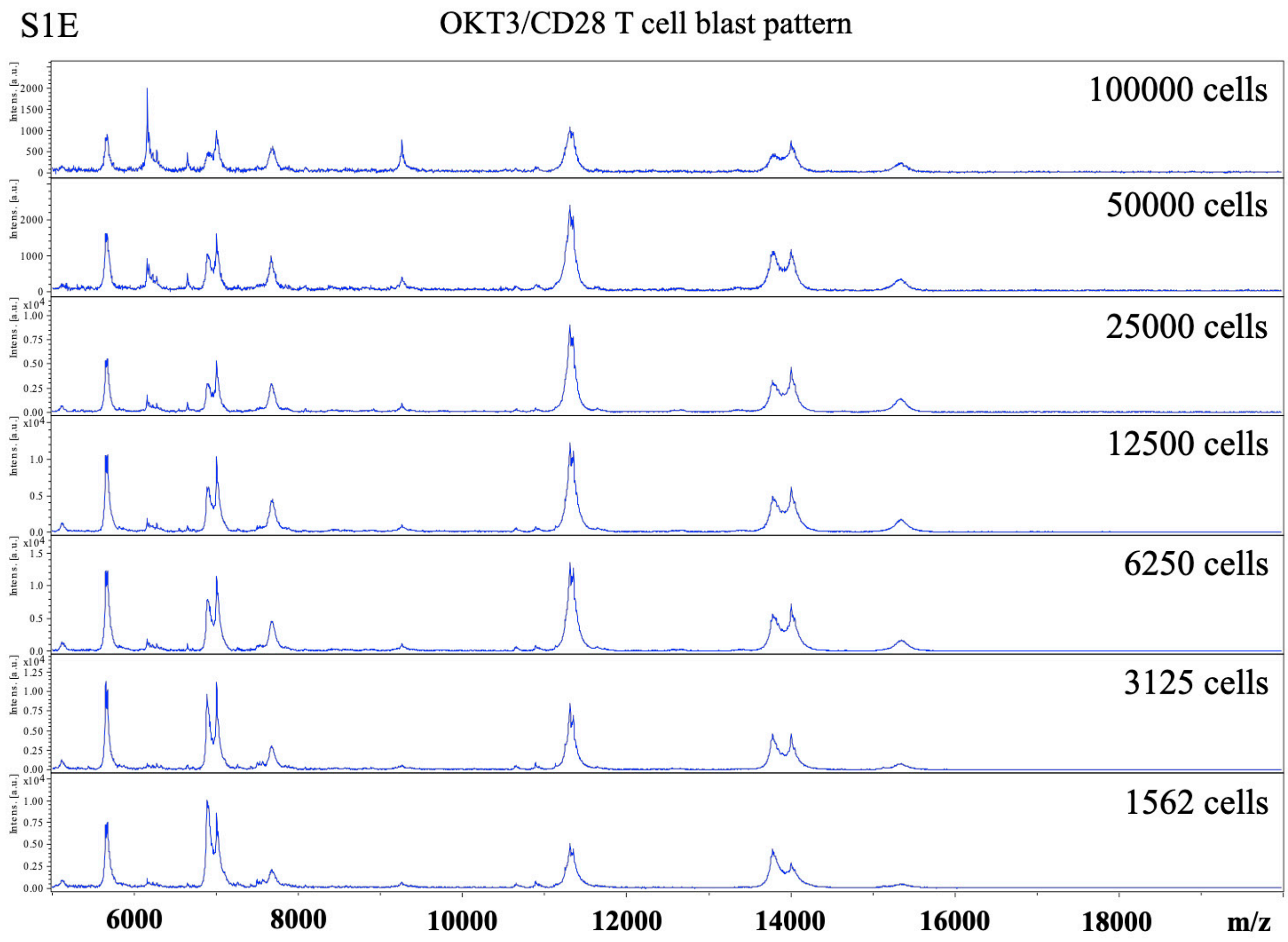
OKT3/CD28 T cell blast pattern
100000 cells
50000 cells
25000 cells
12500 cells
6250 cells
3125 cells
1562 cells
Intens. [a.u.]
x10^4
6000
8000
10000
12000
14000
16000
18000
m/z

S1F

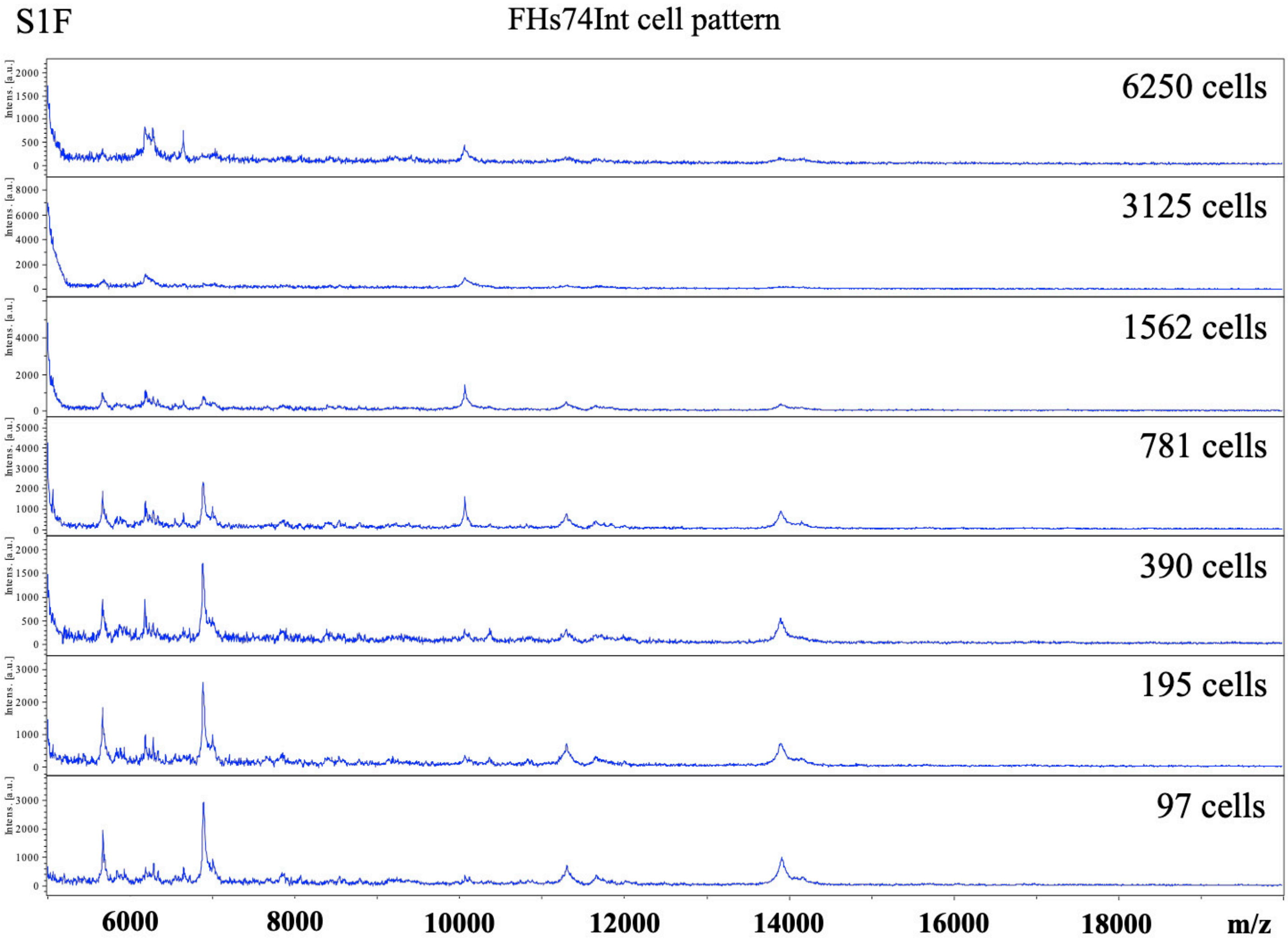
FHs74Int cell pattern
6250 cells
3125 cells
1562 cells
781 cells
390 cells
195 cells
97 cells
Intens. [a.u.]
6000
8000
10000
12000
14000
16000
18000
m/z

S1G

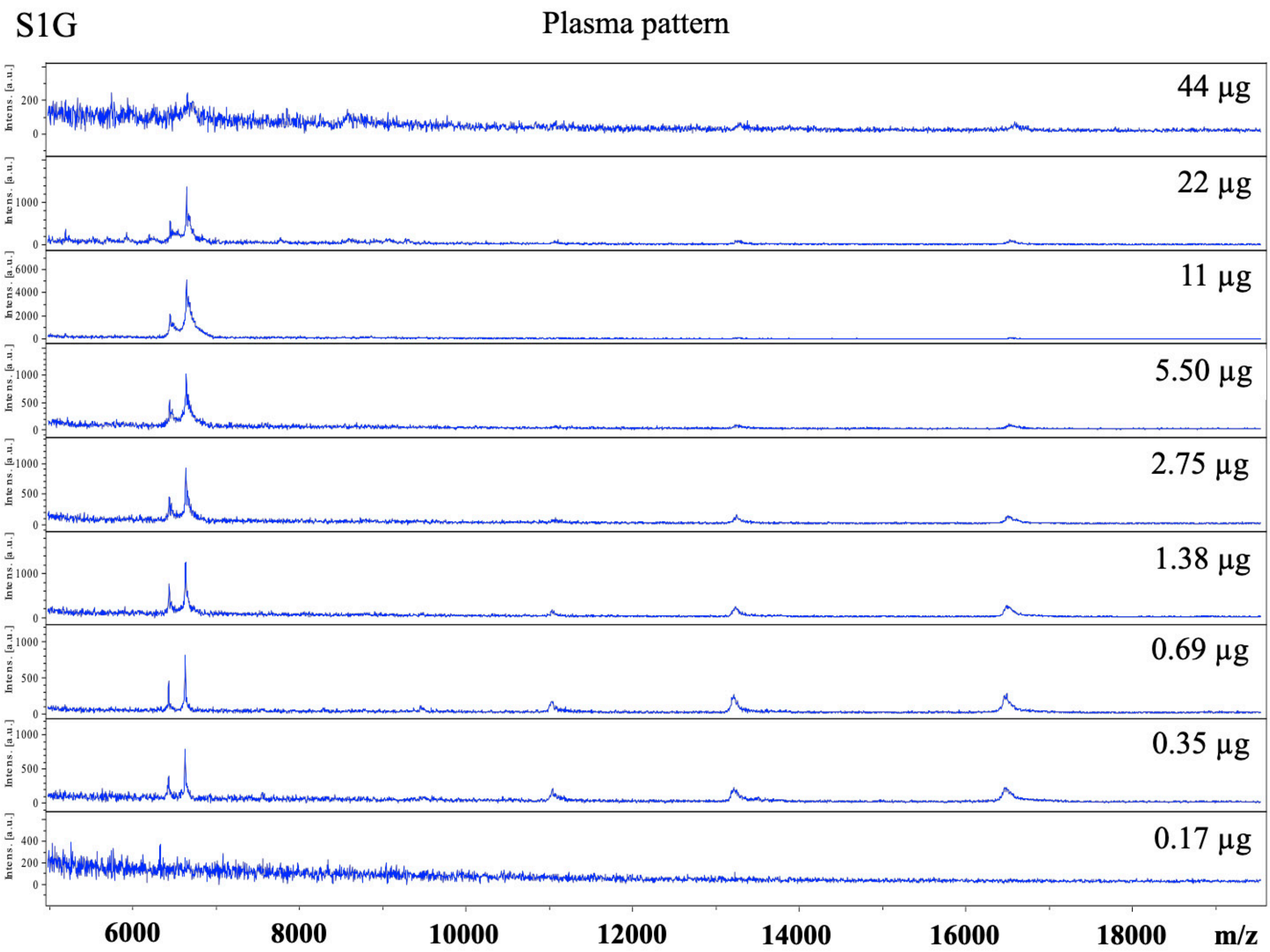

Plasma pattern
Intens. [a.u.]
44 µg
22 µg
11 µg
5.50 µg
2.75 µg
1.38 µg
0.69 µg
0.35 µg
0.17 µg
6000
8000
10000
12000
14000
16000
18000
m/z

S1H

Jurkat cell pattern

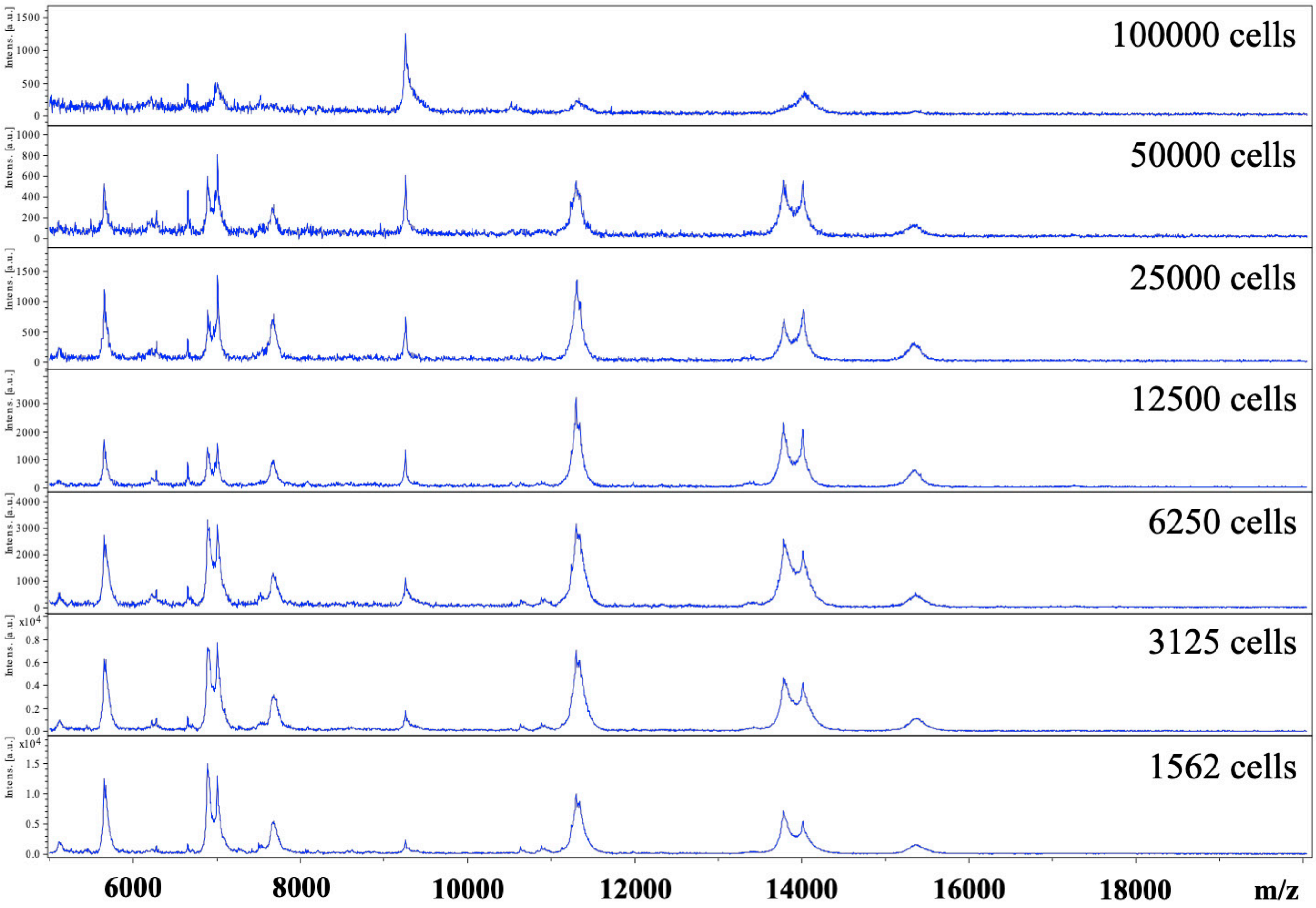

S1I

SK-N-SH cell pattern

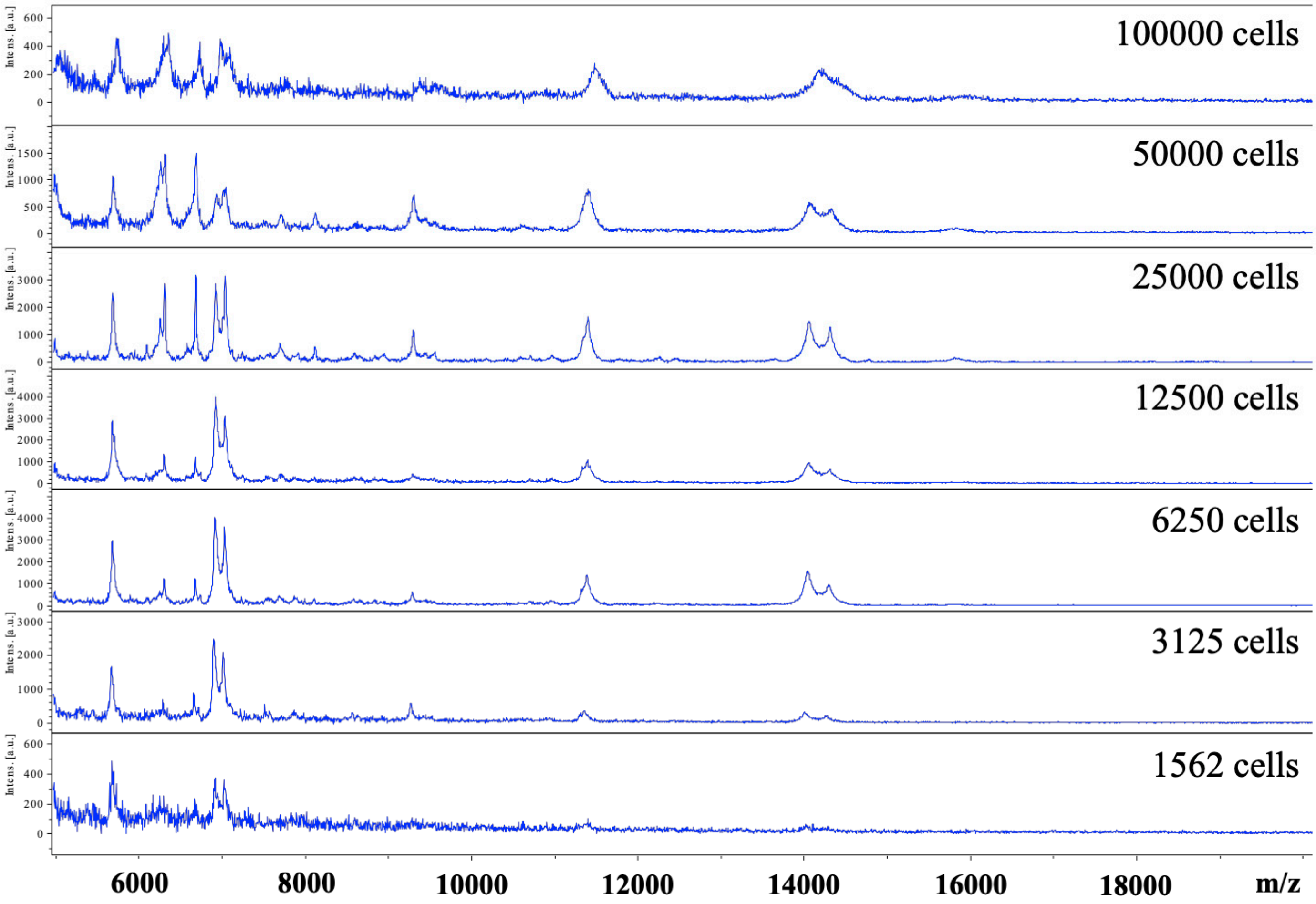

S1J

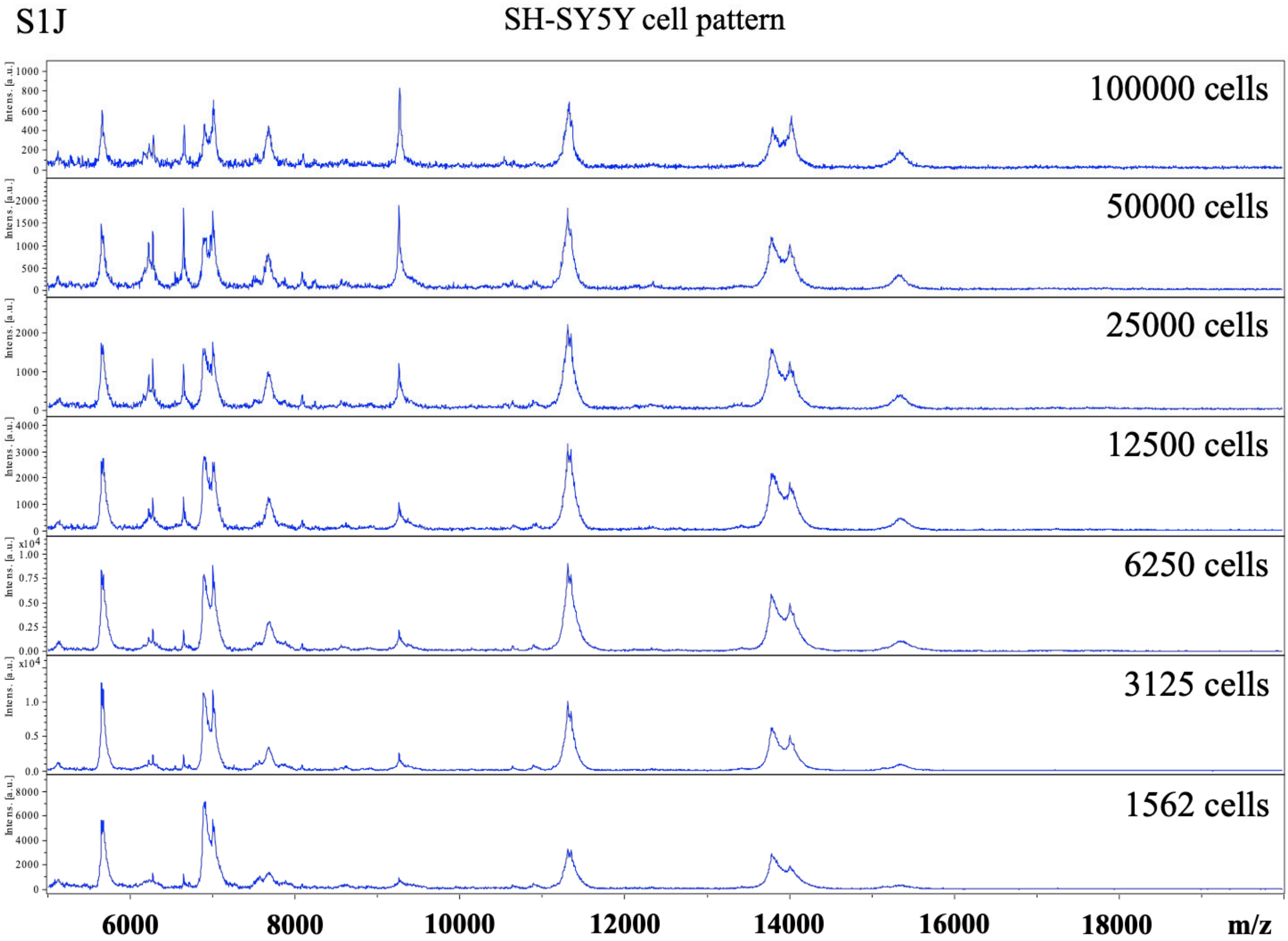
SH-SY5Y cell pattern
100000 cells
50000 cells
25000 cells
12500 cells
6250 cells
3125 cells
1562 cells
Intens. [a.u.]
x10^4
6000
8000
10000
12000
14000
16000
18000
m/z

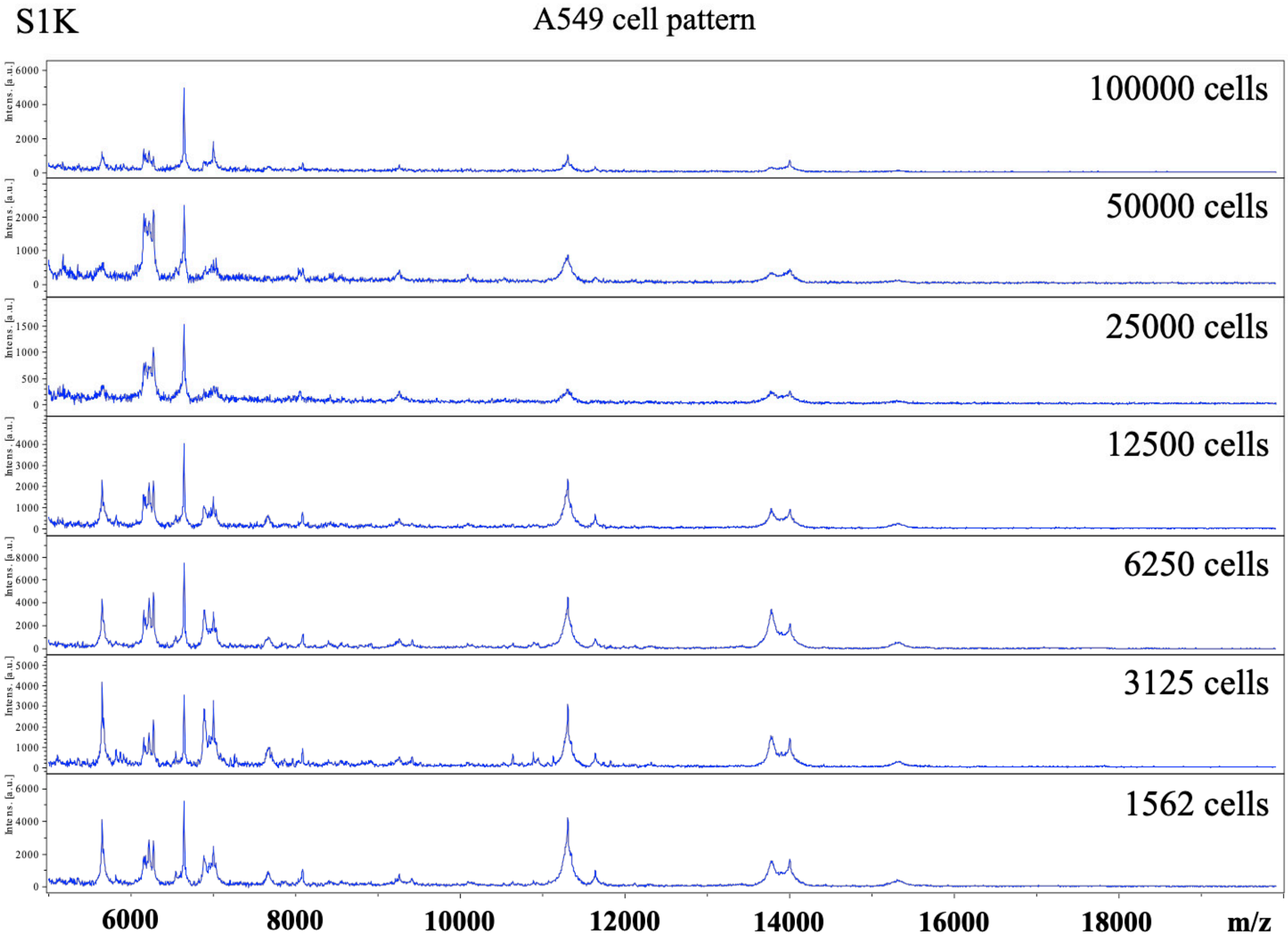
S1K
A549 cell pattern
100000 cells
50000 cells
25000 cells
12500 cells
6250 cells
3125 cells
1562 cells
Intens. [a.u.]
6000
8000
10000
12000
14000
16000
18000
m/z

S1L

A2780 cell pattern

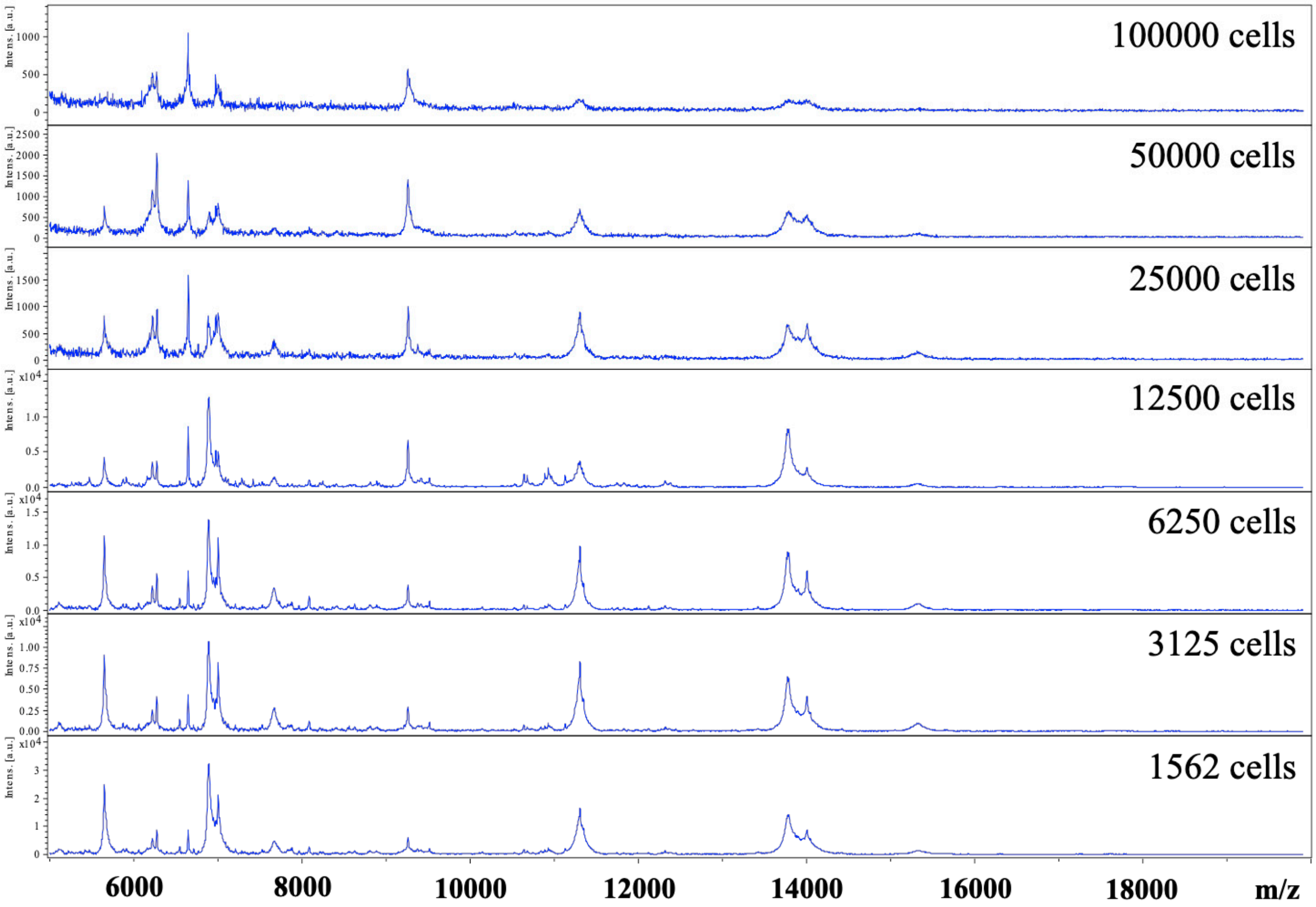

S1M

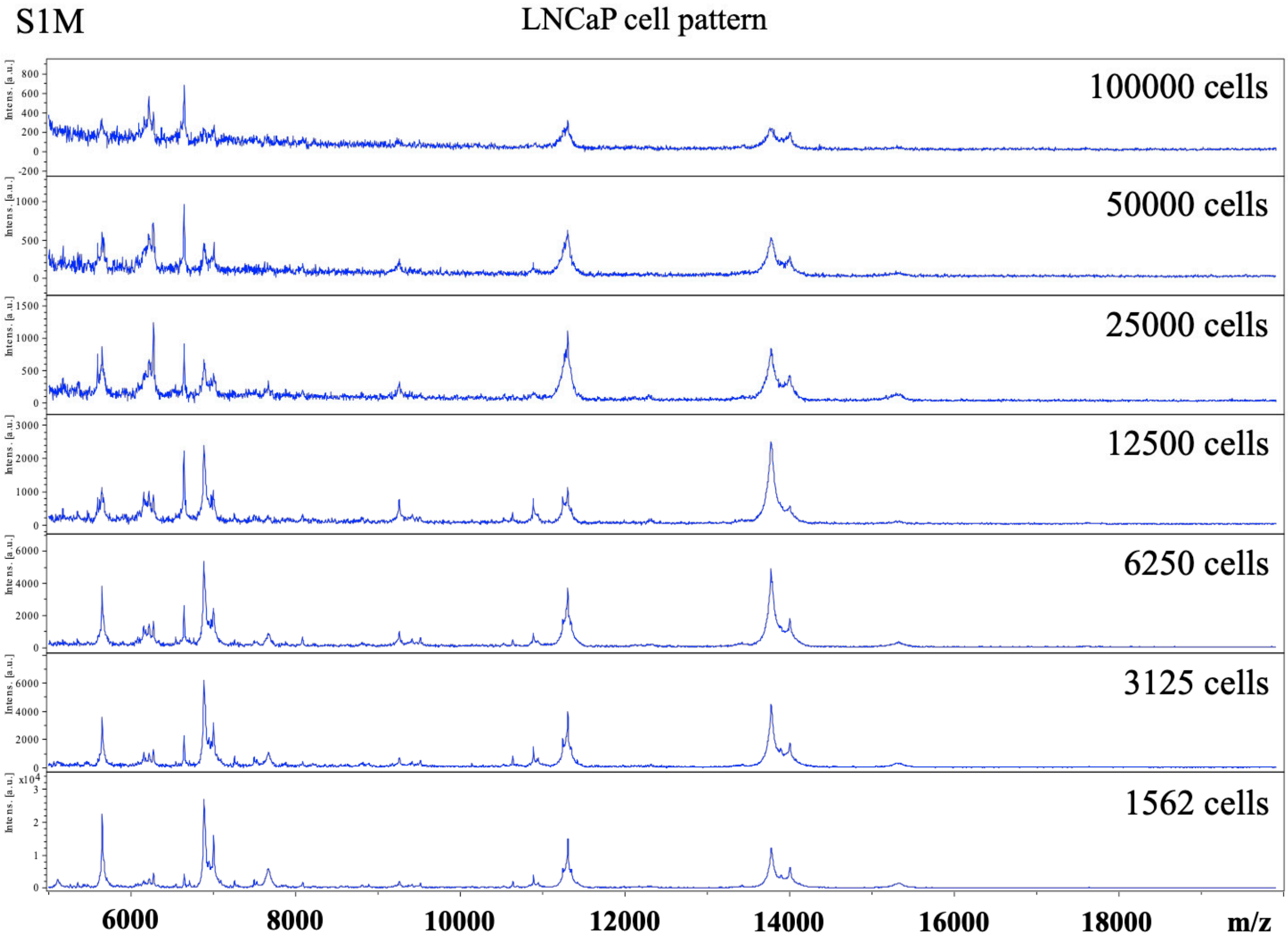
LNCaP cell pattern
100000 cells
50000 cells
25000 cells
12500 cells
6250 cells
3125 cells
1562 cells
Intens. [a.u.]
6000
8000
10000
12000
14000
16000
18000
m/z

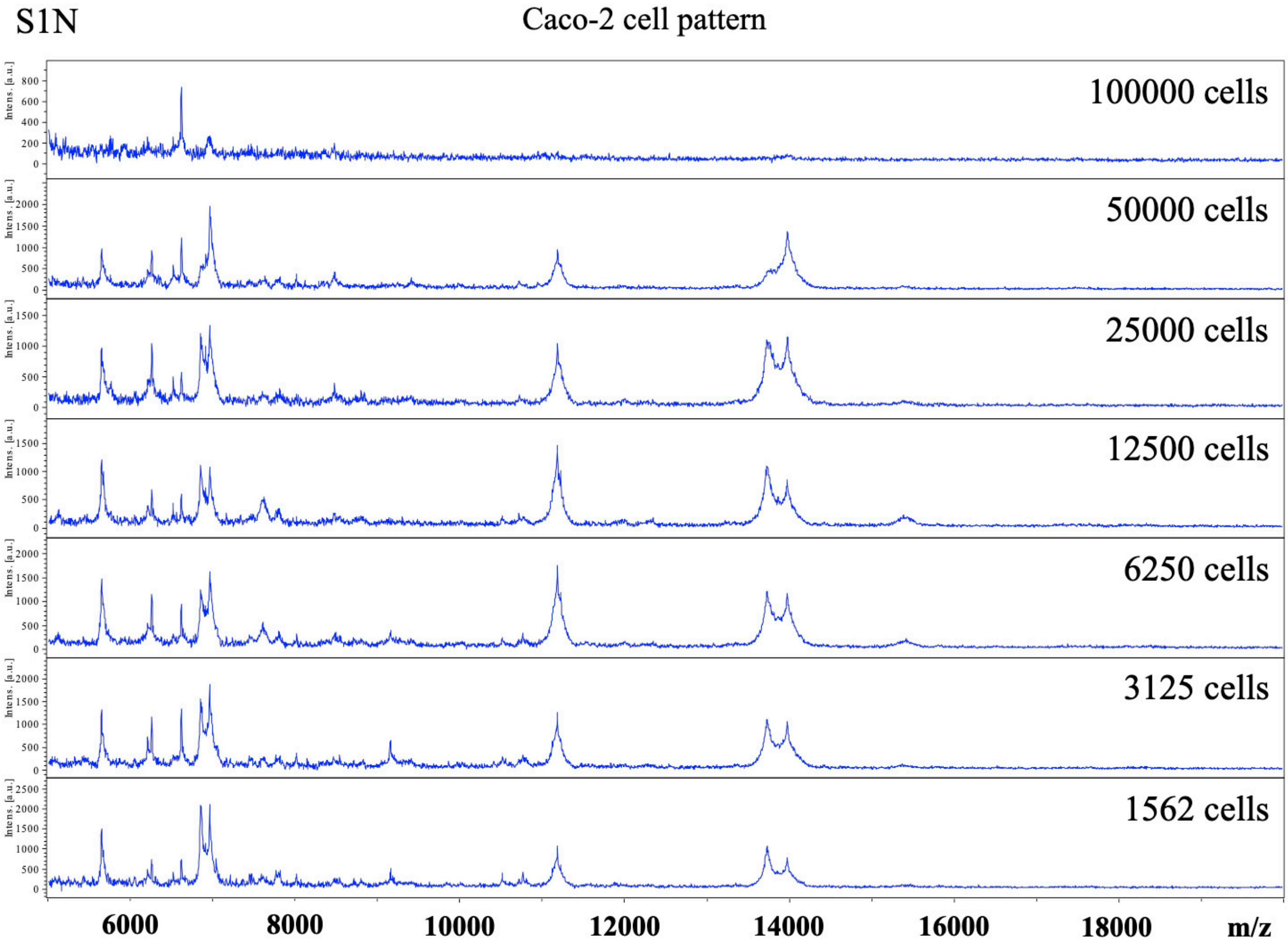

Figure S1. Whole-cell-MSP of normal and cancer cells at various cell dosages. (**A**) RBC; (**B**) WBC; (**C**) PBMC; (**D**) PHA activated T cell; (**E**) OKT/CD28 T cell blast; (**F**) FHs74Int; (**G**) plasma; (**H**) Jurkat; (**I**) SK-N-SH; (**J**) SH-SY5Y; (**K**) A549; (**L**) A2780; (**M**) LNCaP; (**N**) Caco-2.

Table S1. Culture medium for human cell lines in this study.

Name	Tissue	Disease	Culture medium
FHs74Int	Small intestine	Normal	Hybri-Care Medium ATCC 46-X + 90% of 30 ng/mL EGF + 10%FBS
A2780	Ovary	Carcinoma	RPMI-1640+2 mM L-glutamine + 10% FBS + 1× Penicillin/Streptomycin
A549	Lung	Carcinoma	DMEM/HAM'S F-12 + 2 mM·L-glutamine + 10% FBS + 1× Penicillin/Streptomycin
Caco-2	Colon	Colorectal adenocarcinoma	DMEM + 2 mM L-glutamine + 10% FBS + 1× Penicillin/Streptomycin
Jurkat	Peripheral blood	Acute T cell leukemia	RPMI-1640 + 2 mM L-glutamine + 10% FBS + 1× Penicillin/Streptomycin
LNCaP	Prostate	Carcinoma	RPMI-1640 + 2 mM L-glutamine + 10% FBS + 1× Penicillin/Streptomycin
SK-N-SH	Brain	Neuroblastoma	DMEM (1g/L glucose) + 2 mM L-glutamine + 10% FBS + 1× Penicillin/Streptomycin
SH-SY5Y	Bone barrow	Neuroblastoma	DMEM (4.5g/L glucose) + 2 mM L-glutamine + 10% FBS + 1× Penicillin/Streptomycin

Table S2. The mass-to-charge (m/z), intensity, and frequency of main spectrum peaks of normal and cancer cell conditions. Blue, normal conditions. Red, cancer cell conditions.

	Normal		Cancer	
m/z	Intensity	Frequency	Intensity	Frequency
5043.18	15.46	71.7		
5075.46	14.15	66.9		
5093.74	11.08	54.6		
5114.2	10.49	63.3		
5145.94	9.33	49	10.6	60.8
5173.37			10.72	47.6
5183.48	19.18	71.3		
5206.91	12.32	62.9		
5225.35	11.45	48.6	10.11	55.4
5259.13	11.02	45.8		
5313.19	10.63	52.2		
5355.09	13.97	67.7	12.9	83.7
5376.39	11.86	66.9		
5415.29	13.94	57.4		
5421.98			13.31	69.9
5472.78	10.26	49.4		
5585.21	9.91	45.4		
5603.39	11.23	45.4	9.02	51.8
5618.3	13.43	60.2		
5636.54	19.77	49	16.33	92.2
5653.22	57.01	83.7	65.01	92.8
5674.85	37.47	86.1	34.95	100
5691.52	26.22	70.5	17.11	89.2
5707.47			12.51	72.3
5720.72	18.07	69.3	10.27	60.2
5737.01	14.52	58.2		
5755.91	11.04	49.4		
5783.34	10.15	43.4		
5810.49	12.79	50.6		
5830.47	13.07	72.1		
5921.75	13.01	69.3		
5992.05			9.26	60.8
6061.32	11.22	47	8.95	51.8
6159.65	15.64	86.1	12.11	58.4
6177.59	27.36	67.3	20.15	67.5
6195.73	16.91	75.3		
6226.3	24.86	76.9	17.08	90.4
6244.58			15.69	62
6261.99			22.16	57.2
6276.44	39.69	78.9	34.68	83.7
6293.13			12.33	74.7
6298.54	15.63	58.2		
6330.95	12.58	53.4	20.87	66.9
6358.1			26.87	56
6364.12	11.38	54.6		
6383.02			18.26	49.4
6512.09	11.56	46.2	14.75	48.2
6545.47	18.08	58.6	12.26	86.1
6567.28	12.2	49.4		
6591.88	11.24	48.6		
6628.67	23.19	45.4		
6641.78			22.63	67.5
6647.14	38.25	77.3	57.72	51.8
6670.24	20.03	65.7	15.07	81.9

6692.58	12.44	59.4	9.99	57.2
6713.84			8.7	75.3
6728.11	13.14	44.6		
6889.69	30.06	80.1	51.51	95.2
6910.13	21.26	72.5	34.41	84.5
6926.03	24.62	50.6	21.12	71.1
6950.18	13.67	58.2	17.67	89.8
6976.7	13.53	45.8		
6980.44			20.78	95.8
7007.11	25.35	72.9	62.86	98.2
7021.48			33.05	95.2
7036.64	19.17	69.7	21.16	77.7
7057.62	12.95	51.8	12.41	48.2
7080.54	11.49	45.8		
7258.59			8.56	56.6
7527.42			7.09	51.8
7558.62	24.99	50.6		
7624.56			8.18	47
7637.38	11.22	48.2		
7647.25			10.72	63.3
7659.63	10.88	54.6		
7668.73			15.43	48.8
7678.22	11.73	49.4	13.68	75.9
7703.24	10.42	48.6		
7709.9			8.82	49.4
7835.01	10.24	55		
7882.78	12.27	61	8.35	59
7930.28	10.68	45.8		
8087.14	17.18	54.6	12.24	68.7
8558.19			17.45	69.3
8583.8			13.68	47.6
9263.08			28.25	57.8
10844.09			12.01	55.4
10877.55			7.37	54.2
11247.02			15.47	66.9
11268.14	19.6	55.4	19.31	87.3
11289.64	25.21	71.3		
11307.18	44.01	61.4	42.46	93.4
11328.46	29	59.8	25.44	59.6
11348.59	27.72	66.1	27.14	78.9
11366.75			16.37	47.6
11371.86	22.84	53.4		
11383.36			11.76	72.9
13744.32			18.58	61.4
13783.64			28.72	93.4
13788.19			28.63	66.9
13817.97			19.32	68.7
13852.6			13.67	53.6
13908.11			9.88	56
13988.19			23.46	80.7
14014.15			30.39	85.5
14053.31			16.18	74.1
